# Interleukin-10-producing LAG3^+^ regulatory T cells are associated with disease activity and abatacept treatment in rheumatoid arthritis

**DOI:** 10.1186/s13075-017-1309-x

**Published:** 2017-05-16

**Authors:** Shinichiro Nakachi, Shuji Sumitomo, Yumi Tsuchida, Haruka Tsuchiya, Masanori Kono, Rika Kato, Keiichi Sakurai, Norio Hanata, Yasuo Nagafuchi, Shoko Tateishi, Hiroko Kanda, Tomohisa Okamura, Kazuhiko Yamamoto, Keishi Fujio

**Affiliations:** 1 Department of Allergy and Rheumatology, Graduate School of Medicine, the University of Tokyo, 7-3-1 Hongo, Bunkyo-ku, Tokyo, 113-8655 Japan; 2 Department of Immunotherapy Management, Graduate School of Medicine, the University of Tokyo, 7-3-1 Hongo, Bunkyo-ku, Tokyo, 113-8655 Japan; 3 Max Planck-The University of Tokyo Center for Integrative Inflammology, The University of Tokyo, 4-6-1 Komaba, Meguro-ku, Tokyo, 153-8505 Japan

**Keywords:** LAG3, Regulatory T cell, IL-10, Abatacept, Rheumatoid arthritis, Antibody production

## Abstract

**Background:**

Regulatory T cells (Tregs) play a role in the suppression of inflammation in autoimmune diseases, and lymphocyte activation gene 3 (LAG3) was reported as a marker of interleukin (IL)-10-producing Tregs. We aimed to clarify the function of human IL-10-producing CD4^+^CD25^−^LAG3^+^ T cells (LAG3^+^ Tregs) and their association with rheumatoid arthritis (RA).

**Methods:**

LAG3^+^ Tregs of human peripheral blood mononuclear cells (PBMCs) were cultured with B cells and follicular helper T cells to examine antibody suppression effects. The frequency of LAG3^+^ Tregs was evaluated in peripheral blood samples from 101 healthy donors and 85 patients with RA. In patients treated with abatacept, PBMC samples were analyzed before and after treatment. Naive CD4^+^ T cells were sorted and cultured in the presence of abatacept, followed by flow cytometric analysis and function assays.

**Results:**

LAG3^+^ Tregs produced high amounts of IL-10 and interferon-γ, and they suppressed B-cell antibody production more strongly than CD25^+^ Tregs. Cell-to-cell contact was required for the suppressive function of LAG3^+^ Tregs. The frequency of LAG3^+^ Tregs was lower in patients with RA, especially those with higher Clinical Disease Activity Index scores. LAG3^+^ Tregs significantly increased after 6 months of abatacept treatment, whereas CD25^+^ Tregs generally decreased. Abatacept treatment in vitro conferred LAG3 and EGR2 expression on naive CD4^+^ T cells, and abatacept-treated CD4^+^ T cells exhibited suppressive activity.

**Conclusions:**

IL-10-producing LAG3^+^ Tregs are associated with the immunopathology and therapeutic response in RA. LAG3^+^ Tregs may participate in a mechanism for the anti-inflammatory and immune-modulating effects of targeted therapy for costimulation.

**Electronic supplementary material:**

The online version of this article (doi:10.1186/s13075-017-1309-x) contains supplementary material, which is available to authorized users.

## Background

Rheumatoid arthritis (RA) is the most common type of autoimmune disease. It is characterized by synovial inflammation, production of autoantibodies including anticitrullinated protein antibody (ACPA), and destruction of cartilage and bone. With regard to the pathogenesis of RA, dendritic cells and macrophages are initially activated, and they present autoantigens to T cells, leading to the expansion of autoreactive T cells [1]. Autoreactive B cells also play a central role through their differentiation into long-lived memory B cells and autoantibody-producing plasma cells [2]. Activation of autoreactive T cells and B cells further stimulates macrophages to produce proinflammatory cytokines such as tumor necrosis factor-α, interleukin (IL)-1β, and IL-6. Therefore, adaptive immunity mediated by autoreactive T cells and B cells can be one of the most important targets in the therapy of RA [3].

Regulatory T cells (Tregs) constitute a subpopulation of T cells that plays an indispensable role in maintaining tolerance to self-antigens and immunological homeostasis. A lack of Tregs or their dysfunction leads to a breakdown of immunological tolerance to self-antigens and can result in autoimmune diseases [4, 5]. A number of Treg subsets have been identified over the years [6–10], and cluster of differentiation CD4^+^CD25^+^FOXP3^+^ regulatory T cells (CD25^+^ Treg) [6] and CD4^+^ IL-10-producing regulatory T cells (IL-10-producing Tregs) [7] are the best characterized among them.

CD25^+^ Tregs develop mainly in the thymus to specifically express the Forkhead box P3 (*Foxp3*) gene [11]. Scurfy mice, which have a frameshift mutation in the *Foxp3* gene, exhibit severe inflammatory infiltration of the skin and liver [12]. However, many organs, including the central nervous system, joints, and small intestine, remain unaffected in scurfy mice [13]. These results suggest the existence of additional important mechanisms other than CD25^+^ Tregs that support self-tolerance against many organs including joints [14].

IL-10-producing Tregs are characterized by the production of high amounts of IL-10 without FOXP3 expression. IL-10-producing Tregs have been reported to ameliorate experimental autoimmune encephalitis [15] and colitis [7] in mouse models. Thus far, IL-10-producing Tregs have primarily been reported as induced populations in the presence of vitamin D_3_ [16], anti-CD46 antibody [17], rapamycin [18], or IL-27 [19, 20]. This is due in part to the difficulty in identifying naturally occurring IL-10-producing Tregs because of the lack of definitive surface markers. However, recent reports have shown that lymphocyte activation gene 3 (LAG3) protein, a major histocompatibility complex class II-binding CD4 homologue, is expressed on IL-10-producing CD4^+^ T cells and is a candidate phenotypic surface marker for IL-10-producing Tregs [21–23].

We have previously identified murine CD4^+^CD25^−^LAG3^+^ regulatory T cells that produce high amounts of IL-10 and interferon (IFN)-γ, lack Foxp3 expression, and suppress B-cell antibody production [21, 23]. They are regulated by early growth response gene 2 (Egr2), which is important for the maintenance of T-cell anergy by negatively regulating T-cell activation [24]. We therefore hypothesized that human CD4^+^CD25^−^LAG3^+^ T cells might have the same functions as those in mice and that they might be associated with human autoimmune diseases. We aimed to characterize CD4^+^CD25^−^LAG3^+^ T cells in healthy and autoimmune states and to determine the impact of abatacept treatment that targets T-cell responses.

## Methods

### Blood samples and clinical data

All clinical investigations conformed to the Declaration of Helsinki principles and were approved (10154 and G3582) by the ethics committee of the University of Tokyo. Peripheral blood mononuclear cells (PBMCs) were obtained from 101 self-reported screened healthy donors and 85 patients with RA who fulfilled the 1987 American College of Rheumatology revised criteria or the 2010 American College of Rheumatology/European League Against Rheumatism classification criteria. Moreover, PBMCs were taken from four healthy donors vaccinated with a seasonal inactivated influenza virus in 2013. Clinical characteristics and laboratory data were documented on the day of sample collection. All subjects provided written informed consent.

### Cell isolation and flow cytometry

**Fig. 1:**
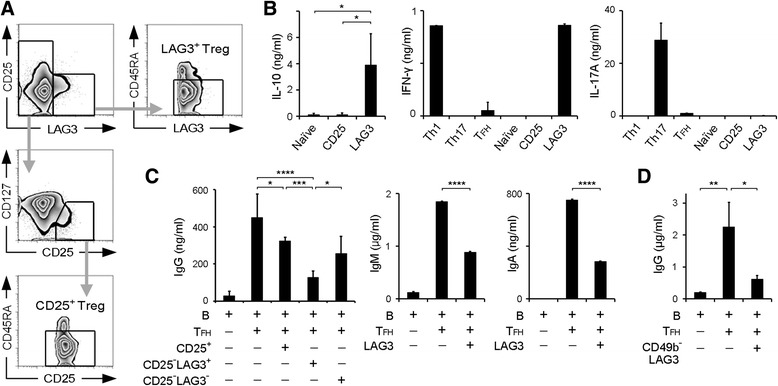
Human CD4^+^CD25^−^LAG3^+^ T cells suppressed antibody production. **a** Gating strategy of fluorescence-activated cell sorting analysis for CD4^+^CD25^−^LAG3^+^CD45RA^−^ T cells (LAG3^+^ Tregs) and CD4^+^CD25^+^CD127^dim^CD45RA^−^ T cells (CD25^+^ Tregs). **b** IL-10, IFN-γ, and IL-17A protein levels in the culture supernatants after 3 days of incubation of the indicated T-cell subsets was determined by ELISA (*n* = 6). **c** B cells and T_FH_ cells were cocultured with the indicated subsets for 12 days in the presence of SEB stimulation, and total IgG, IgM, and IgA production was determined by ELISA (*n* = 6). **d** B cells and T_FH_ cells were cocultured with CD4^+^CD25^−^LAG3^+^CD49b^−^ T cells for 12 days in the presence of SEB stimulation, and total IgG was determined by ELISA (*n* = 3). All error bars represent SD. * *P* < 0.05; ** *P* < 0.01; *** *P* < 0.001; **** *P* < 0.0001. All *P* values were calculated by one-way analysis of variance and Tukey’s multiple-comparisons test. *CD* Cluster of differentiation, *ELISA* Enzyme-linked immunosorbent assay, *IFN-γ* Interferon-γ, *Ig* Immunoglobulin, *IL* Interleukin, *LAG3* Lymphocyte activation gene 3, *RA* Rheumatoid arthritis, *SEB* Staphylococcal enterotoxin B, *T*_*FH*_ Follicular helper T cell, *Treg* Regulatory T cell

PBMCs were isolated from whole blood by Ficoll-Paque Plus (GE Healthcare Life Sciences, Pittsburgh, PA, USA) gradient separation. Fc receptor binding inhibitor (eBioscience, San Diego, CA, USA) was added to the isolated PBMCs. They were stained with the following monoclonal antibodies (mAbs) for 20 minutes: Alexa Fluor 488 anti-C-X-C chemokine receptor type 5 (anti-CXCR5; RF8B2), phycoerythrin (PE) anti-C-C chemokine receptor type 6 (anti-CCR6; 11A9), Brilliant Violet 412 anti-CXCR3 (1C6), and V500 anti-CD4 (RPA-T4) from BD Biosciences (San Jose, CA, USA); Alexa Fluor 488 anti-CD49b (AK7), peridinin-chlorophyll protein (PerCP)-Cy5.5 anti-CD3 (UCHT1), PerCP-Cy5.5 anti-CCR7 (G043H7), allophycocyanin anti-CD19 (HIB19), allophycocyanin-Cy7 anti-CD45RA (HI100), and Brilliant Violet 421 anti-CD25 (BC96) from BioLegend (San Diego, CA, USA); PE-Cy7 anti-CD127 (eBioRDR5) from eBioscience; and PE anti-LAG3 polyclonal antibody (FAB2319P, 4:100) from R&D Systems (Minneapolis, MN, USA). All cell subsets were analyzed and sorted by eight-color MoFlo XDP (Beckman Coulter Life Sciences, Indianapolis, IN, USA). Each subset was defined as having the following immunophenotype: naive CD4^+^ T cells were CD4^+^CD45RA^+^CD25^−^LAG3^−^CCR7^+^ (Fig. 1) or CD4^+^CD45RA^+^ (Fig. 4); follicular helper T (T_FH_) cells were CD4^+^CD45RA^−^CXCR5^+^CD25^−^LAG3^−^; CD25^+^ Tregs were CD4^+^CD45RA^−^CD25^+^LAG3^−^CD127^dim^; type 1 helper T (Th1) cells were CD4^+^CD45RA^−^CXCR3^+^CCR6^−^; Th2 cells were CD4^+^CD45RA^−^CXCR3^−^CCR6^−^; Th17 cells were CD4^+^CD45RA^−^CXCR3^−^CCR6^+^; LAG3^+^ Tregs were CD4^+^CD45RA^−^CD25^−^LAG3^+^; and B cells were CD3^−^CD19^+^. Data were analyzed using FlowJo software (FlowJo, Ashland, OR, USA).

### Measurement of cytokines by enzyme-linked immunosorbent assay

CD4^+^ T cells (2 × 10^4^ cells/well) were stimulated with plate-bound anti-CD3 mAb (5.0 μg/ml, UCHT1; R&D Systems) and anti-CD28 mAb (1.0 μg/ml, 37407; R&D Systems) in flat-bottomed 96-well plates for 3 days. Concentrations of IL-10, IFN-γ, and IL-17A in the supernatants were measured using enzyme-linked immunosorbent assay (ELISA) kits according to the manufacturer’s protocol (OptEIA Human IL-10 ELISA Kit II; BD Biosciences; Human IFN-γ ELISA Ready-SET-Go! and Human IL-17A ELISA Ready-SET-Go!; eBioscience). Cells were cultured in Gibco RPMI 1640 medium (Invitrogen, Paisley, UK) supplemented with 10% FBS (Equitech-Bio, Kerrville, TX, USA), 100 μg/ml l-glutamine, 100 U/ml penicillin, 100 μg/ml streptomycin, and 50 μM 2-mercaptoethanol (Life Technologies, Carlsbad, CA, USA).

### B-cell and T-cell coculture

Each CD4^+^ T cell subset (1 × 10^5^ cells/well) was cocultured with B cells (1 × 10^5^ cells/well) and T_FH_ cells (5 × 10^4^ cells/well) in round-bottomed 96-well plates. Recombinant staphylococcal enterotoxin B (SEB) (2 μg/ml; Toxin Technology, Sarasota, FL, USA) was added to stimulate B cells. Blocking antihuman IL-10 mAb (25209; R&D Systems), blocking antihuman Fas ligand mAb (100410; R&D Systems), or mouse immunoglobulin G1κ (IgG1κ) isotype control (eBioscience) was added at a concentration of 10 μg/ml on the first day of the coculture in the same experimental system as above. To assess cell-cell contact, a coculture experiment was performed using a Transwell plate (HTS Transwell-96 System [3381]; Corning Life Sciences, Acton, MA, USA) with the following cells: B cells, 3 × 10^5^ cells/well; T_FH_ cells, 2 × 10^5^ cells/well; and CD4^+^CD25^−^LAG3^+^CD45RA^−^ T cells, 1 × 10^5^ cells/well. The concentrations of IgG, IgA, and IgM in the 12-day culture supernatant were measured by ELISA (Human IgG ELISA Quantitation Set, Human IgA ELISA Quantitation Set, and Human IgM ELISA Quantitation Set; Bethyl Laboratories, Montgomery, TX, USA).

### Culture in the presence of abatacept

Antigen-presenting cells (APCs) were sorted as CD3^−^ cells and irradiated with 30 Gy. Abatacept was purchased from Bristol-Myers Squibb (Princeton, NJ, USA) and used at a final concentration of 10 μg/ml. Human IgG1 Fc (R&D Systems) was added as a control. Cultured cells were analyzed and sorted as 7-amino actinomycin D (7-AAD)-negative cells at day 4 (BioLegend). Sorted 7-AAD-negative CD4^+^ T cells were cultured with B cells and T_FH_ cells in the aforementioned system to investigate the suppressive function of the cells induced by abatacept.

### Quantitative real-time polymerase chain reaction

Total RNA was isolated using RNeasy Micro Kit (QIAGEN, Valencia, CA, USA) according to the manufacturer’s protocol. Complementary DNA was synthesized with SuperScript III Reverse Transcriptase (Life Technologies) and oligo(dT) primers, and it was mixed with SYBR Green Master Mix (Life Technologies). The real-time polymerase chain reaction (PCR) primer pairs were as follows: human EGR2 sense: 5′-TGGAGAGAAGAGGTCGTTGG-3′ and antisense: 5′-CTGGATGAGGCTGTGGTTG-3′; FAS sense: 5′-TGCAGAAGATGTAGATTGTGTGATGA-3′ and antisense: 5′-GGGTCCGGGTGCAGTTTATT-3′, and CD247 sense: 5′-GGGAGAATGATGGATGTGAA-3′ and antisense: 5′-CCGATGAACCCCTAAACCA-3′. Quantitative PCR was conducted using an iCycler PCR system (Bio-Rad Laboratories, Hercules, CA, USA). The expression of each gene was normalized to the glyceraldehyde 3-phosphate dehydrogenase housekeeping gene (*GAPDH*) (forward: 5′-GAAGGTGAAGGTCGGAGTC-3′ and reverse: 5′-GAAGATGGTGATGGGATTTC-3′).

### Statistical analysis

Data are presented as mean ± SD. Statistical differences between groups were evaluated using an unpaired two-tailed Student’s *t* test, the Mann-Whitney *U* test, or the Wilcoxon signed-rank test for comparing two groups, and one-way analysis of variance or the Kruskal-Wallis test was used to compared three or more groups. *P* values less than 0.05 were considered statistically significant (* *P* < 0.05; ** *P* < 0.01; *** *P* < 0.001; **** *P* < 0.0001). Calculations were conducted using Prism version 5.03 software (GraphPad Software Inc., La Jolla, CA, USA). To compare the frequency of Tregs in patients, a statistical analysis was conducted using R version 3.1.2 software (R Foundation for Statistical Computing, Vienna, Austria).

## Results

### Human CD4^+^CD25^−^LAG3^+^CD45RA^−^ T cells produce IL-10 and suppress antibody production

In human PBMCs, we identified CD4^+^CD25^−^LAG3^+^ T cells that were negative for Foxp3. Confirmation of Foxp3 expression and suppressive activity of control CD4^+^CD25^+^ Tregs indicated the adequacy of gating strategies (Fig. 1a and Additional file 1: Figures S1 and S2). Upon T-cell receptor (TCR) stimulation, CD4^+^CD25^−^LAG3^+^ T cells produced high amounts of IL-10, amounts of IFN-γ as much as Th1 cells, and no IL-17A (Fig. 1b), consistent with the results for murine CD4^+^CD25^−^LAG3^+^ Tregs [21]. Intracellular staining revealed that more LAG3^+^ T cells (mean 14.2%) expressed IL-10 than naive CD4^+^ T cells (mean 4.8%) (Additional file 1: Figure S3). Given that murine CD4^+^CD25^−^LAG3^+^ Tregs efficiently control B-cell functions, we cocultured these cells with B cells and T_FH_ cells and measured IgG in the supernatants. When SEB was used to imitate the antigen-specific interaction between B cells and T_FH_ cells, we found that CD4^+^CD25^−^LAG3^+^ T cells significantly suppressed IgG production by B cells (Fig. 1c). Moreover, CD4^+^CD25^−^LAG3^+^ T cells also suppressed IgM and IgA (Fig. 1c), which indicated that CD4^+^CD25^−^LAG3^+^ T cells inhibited immunoglobulin production instead of inhibiting class switching. In a previous study, LAG3^+^CD49b^+^ T cells were reported to have the suppressive function of T cells among IL-10-producing Tregs [22]. Therefore, we assessed whether CD49b was required for antibody suppression by CD4^+^CD25^−^LAG3^+^ T cells and found that CD49b^−^CD4^+^CD25^−^LAG3^+^ T cells significantly suppressed antibody production (Fig. 1d). Taken together, these results showed that CD4^+^CD25^−^LAG3^+^ T cells in human PBMCs produced high levels of IL-10 and suppressed B-cell antibody production, regardless of CD49b expression.

### The suppressive function of CD4^+^CD25^−^LAG3^+^ T cells requires cell-to-cell contact

**Fig. 2:**
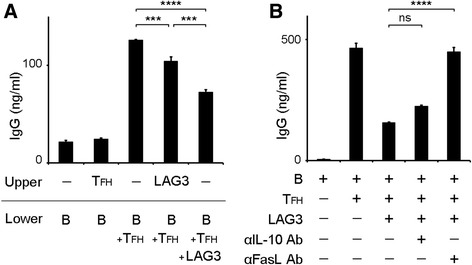
LAG3^+^ Tregs suppressed B-cell antibody production independent of IL-10. **a** B cells and T_FH_ cells were cocultured with LAG3^+^ Tregs under staphylococcal enterotoxin B stimulation in Transwells for 12 days. The indicated subsets were placed in upper and lower chambers as described. Total IgG production was determined by ELISA (*n* = 3). **b** Anti-IL-10 antibody and anti-FasL antibody were added to the same coculture system as in (**a**). Total IgG production was determined by ELISA (*n* = 3). All error bars represent SD. *** *P* < 0.001; **** *P* < 0.0001. All P values were calculated by one-way analysis of variance and Tukey’s multiple-comparison test. *Ab* Antibody, *ELISA* Enzyme-linked immunosorbent assay, *FasL* Fas ligand, *Ig* Immunoglobulin, *IL* Interleukin, *LAG3* Lymphocyte activation gene 3, *T*_*FH*_ Follicular helper T cell, *Treg* Regulatory T cell

We conducted experiments using Transwell plates and found that the suppressive function of CD4^+^CD25^−^LAG3^+^ T cells was abrogated in the absence of direct contact with B cells or T_FH_ cells (Fig. 2a). Among interactions between cell surface molecules, Fas–Fas ligand interaction is associated with antibody suppression of murine LAG3^+^ Tregs [23]. In a blocking experiment, the addition of anti-Fas ligand antibody abrogated the suppressive function of these cells, whereas anti-IL-10 antibody did not affect this suppression (Fig. 2b). This suggests that CD4^+^CD25^−^LAG3^+^ T cells exerted their suppressive function in a manner requiring Fas–Fas ligand interaction rather than IL-10.

### The frequency of LAG3^+^ Tregs was decreased in patients with rheumatoid arthritis and significantly increased with abatacept treatment

**Fig. 3:**
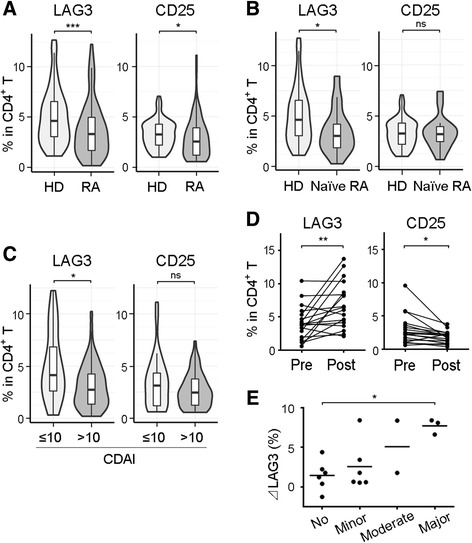
Frequencies of LAG3^+^ Tregs and CD25^+^ Tregs in patients with RA and in healthy volunteers, as well as the effect of abatacept treatment. **a** and **b** PBMCs were taken from healthy donors (HD; *n* = 101) and patients with RA (*n* = 85, including 16 nontreated patients [naive RA]), and Treg subsets were assessed. **c** Patients with RA were divided into two groups according to Clinical Disease Activity Index (CDAI). Treg subsets were assessed (CDAI ≤10, *n* = 30; CDAI >10, *n* = 55). **d** Treg subsets were analyzed immediately before and 6 months after abatacept treatment (*n* = 18). **e** Changes of percentages of LAG3^+^ Tregs in CD4^+^ T cells (ΔLAG3) were evaluated in accordance with treatment response to abatacept (*n* = 18). No response was defined as less than 50% improvement from baseline CDAI. Minor response was defined as at least a 50% improvement from baseline CDAI. Moderate response was defined as at least a 70% improvement from baseline CDAI. Major response was defined as at least an 85% improvement from baseline CDAI. *, *P* < 0.05; **, *P* < 0.01; ***, *P* < 0.001. Statistics: (**a**–**c**) unpaired two-tailed Student’s *t* tests, (**d**) Wilcoxon signed-rank test, (**e**) Kruskal-Wallis test and Dunn’s multiple-comparisons test. *CD* Cluster of differentiation, *LAG3* Lymphocyte activation gene 3, *ns* Not significant, *PBMC* Peripheral blood mononuclear cell, *RA* Rheumatoid arthritis, *Treg* Regulatory T cell

We analyzed 101 healthy subjects and 85 patients with RA to assess LAG3^+^ Treg frequency (Table 1). The frequency of LAG3^+^ Tregs was significantly lower in patients with RA than in healthy donors (3.76 ± 2.72% in RA vs 5.07 ± 2.78% in healthy donors, *P* < 0.001) (Fig. 3a). Moreover, the frequency of LAG3^+^ Tregs in untreated patients with RA was significantly lower than that of healthy donors (*P* < 0.05) (Fig. 3b). Whereas the frequency of CD25^+^ Tregs in patients with RA was significantly lower than that in healthy donors (*P* < 0.05) (Fig. 3a), in the patients with untreated RA, the frequency of CD25^+^ Tregs was not different from that in healthy donors (Fig. 3b). Moreover, LAG3^+^ Tregs demonstrated a significant decrease in patients with higher disease activity (*P* < 0.05) (Fig. 3c), whereas the frequencies of CD25^+^ Tregs were similar regardless of disease states. However, there were not significant correlations between ACPA or rheumatoid factor (RF) titers and frequencies of CD25^+^ Tregs or LAG3^+^ Tregs (Additional file 1: Figure S4). To investigate the effect of RA treatment for LAG3^+^ Tregs, we focused on patients undergoing treatment with abatacept (Table 1). Abatacept treatment significantly increased the frequency of LAG3^+^ Tregs and significantly decreased the frequency of CD25^+^ Tregs (Fig. 3d). Moreover, we divided abatacept-treated patients into four groups (no response, minor response, moderate response, and major response) following the definition of treatment response based on Clinical Disease Activity Index [25, 26]. We analyzed the correlation between treatment response and increase in LAG3^+^ Tregs and found that LAG3^+^ Tregs significantly increased in the patients with a major response to abatacept compared with the patients with no response (Fig. 3e). The same result was obtained from the analysis using the definition of treatment response based on Disease Activity Score in 28 joints based on erythrocyte sedimentation rate (Additional file 1: Figure S5). Taken together, LAG3^+^ Tregs were present at a lower frequency in patients with RA than in healthy donors. Among patients with RA, the frequency of LAG3^+^ Tregs was inversely correlated with disease activity. Moreover, abatacept increased the frequency of LAG3^+^ Tregs in patients with RA.

**Table 1:** Baseline demographics and clinical characteristics

	RA (*n* = 85)	Naive RA (*n* = 16)	RA before abatacept (*n* = 18)
Female sex, *n* (%)	67 (79)	10 (63)	15 (83)
Age, years	58.9 (13.1)	55.9 (12.2)	62.7 (13.2)
Disease duration, months	139.4 (153.9)	6.7 (8.3)	187.5 (144.2)
DAS28-CRP	3.7 (1.5)	4.1 (1.6)	4.2 (1.3)
DAS28-ESR	4.4 (1.6)	4.9 (1.6)	4.9 (1.2)
CDAI	16.6 (12.1)	21.6 (14.5)	21.4 (11.7)
Rheumatoid factor, % positive	74 (87)	15 (94)	15 (83)
Anti-CCP antibody, % positive	72 (85)	13 (81)	18 (100)
Levels of CRP, mg/dl	1.81 (3.24)	3.40 (5.30)	1.54 (2.85)
Treatment naive, *n* (%)	16 (19)	16 (100)	0 (0)
Treatment with MTX, *n* (%)	41 (48)	0 (0)	10 (56)
Treatment with biologics, *n* (%)	19 (22)	0 (0)	5 (28)
TNF-α inhibitor	8 (9)	0 (0)	3 (17)
Tocilizumab	5 (6)	0 (0)	2 (11)
Abatacept	6 (7)	0 (0)	0 (0)

*Abbreviations: RA* Rheumatoid arthritis, *DAS28* Disease Activity Score in 28 joints, *CRP* C-reactive protein, *ESR* Erythrocyte sedimentation rate, *CDAI* Clinical Disease Activity Index, *CCP* Cyclic citrullinated peptide, *MTX* Methotrexate, *TNF* Tumor necrosis factor

Data are presented as mean (SD), unless otherwise specified

### Abatacept facilitates the differentiation of LAG3^+^ Treg-like cells

**Fig. 4:**
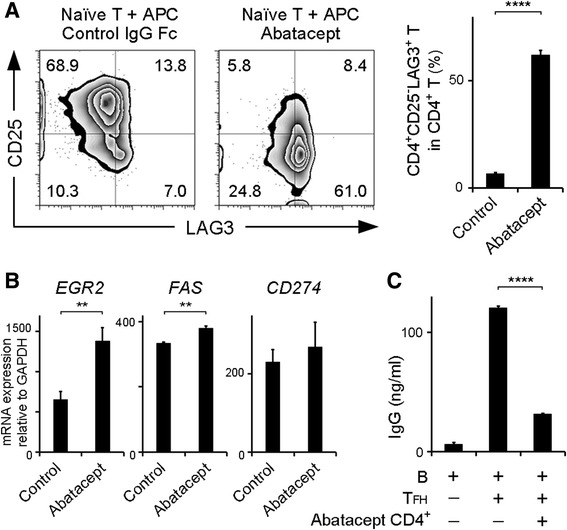
Effect of abatacept on the CD4^+^CD25^−^LAG3^+^ T-cell subset in vitro. **a** Naive CD4^+^ T cells and antigen-presenting cells (APCs) taken from healthy donors were cultured with anti-CD3 antibody in the presence of abatacept, and FACS was conducted on day 4. Representative FACS data from three independent experiments are shown. Percentages of CD4^+^CD25^−^LAG3^+^ T cells in CD4^+^ T cells are presented in the bar graph (*n* = 3). **b** Quantitative real-time polymerase chain reaction analysis of *EGR2*, *FAS*, and *CD274* (*PDL1*) mRNA expression in the cells that were induced from naive CD4^+^ T cells in the presence of abatacept (*n* = 3). **c** Sorted 7-amino actinomycin D-negative CD4^+^ T cells were cultured with B cells and T_FH_ cells under staphylococcal enterotoxin B stimulation for 12 days, and total IgG production was determined by ELISA (*n* = 3). All error bars represent SD. ** *P* < 0.001; **** *P* < 0.0001. All *P* values were calculated by unpaired two-tailed Student’s *t* test. *CD* Cluster of differentiation 4, *EGR2* Early growth response gene 2, *FACS* Fluorescence-activated cell sorting, *GAPDH* Glyceraldehyde 3-phosphate dehydrogenase, *IgG* Immunoglobulin G, *LAG3* Lymphocyte activation gene 3, *mRNA* Messenger RNA, *PDL1* Programmed death-ligand 1, *T*_*FH*_ Follicular helper T cell

Naive CD4^+^ T cells from healthy donors were cultured in the absence or presence of abatacept with irradiated APCs and anti-CD3 antibody for 4 days. When naive CD4^+^ T cells were cultured with abatacept, CD4^+^CD25^−^LAG3^+^ T cells were more strongly induced than naive CD4^+^ T cells cultured without abatacept (*P* < 0.0001) (Fig. 4a). In contrast, the CD4^+^ T cells expressing CD25 decreased in the presence of abatacept (Fig. 4a). Moreover, the expression levels of *EGR2* and *FAS* were increased in the abatacept-treated group (*P* < 0.01) (Fig. 4b). The expression of *CD274* (programmed death-ligand 1 [*PD*-*L1*]) had a tendency to increase in the abatacept-treated group. Egr2 is a key transcription factor that regulates LAG3^+^ Tregs [21]. Both Fas [23] and PD-L1 [27] were reported to be involved in LAG3^+^ Treg suppression ability. Furthermore, abatacept-induced CD4^+^ T cells significantly suppressed antibody production compared with the positive control (*P* < 0.0001) (Fig. 4c). Taken together, these results indicated that abatacept facilitated the differentiation of Egr2-expressing LAG3^+^ Treg-like cells from naive CD4^+^ T cells.

### Active immunization increases Th1 cells and decreases LAG3^+^ Tregs

**Fig. 5:**
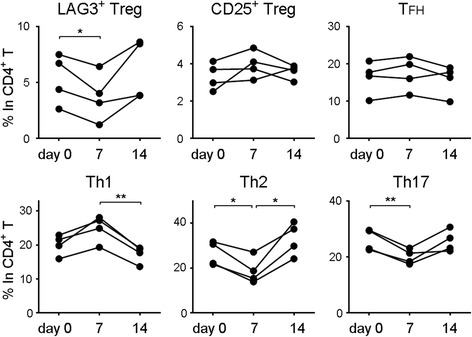
Successive changes of CD4^+^ T-cell subsets after vaccination. Four healthy donors were inoculated with influenza vaccine. The indicated T-cell subsets were evaluated on day 0 (before vaccination), day 7, and day 14. * *P* < 0.05; ** *P* < 0.01. *P* values were calculated by one-way analysis of variance and Tukey’s multiple-comparisons test. *CD* Cluster of differentiation, *LAG3* Lymphocyte activation gene 3, *ns* Not significant, *T*_*FH*_ Follicular helper T cell, *Th* Helper T cell, *Treg* Regulatory T cell

At this point, it was possible that immunological stimulation in RA may have contributed to the decrease of LAG3^+^ Tregs. We addressed whether LAG3^+^ Tregs exhibited numerical alteration in response to active immunization. Vaccination triggers an immune response in human bodies, preparing Th1 cells and memory B cells to respond quickly to previously encountered antigens. To investigate whether LAG3^+^ Tregs were affected by an immune response against foreign antigens, we inoculated four healthy subjects with seasonal influenza vaccine, which is a hemagglutinin fraction of influenza virus mixed strains grown in fertilized chicken eggs without adjuvant, and evaluated T-cell subsets in PBMCs at days 0, 7, and 14. All four subjects showed an increase in the frequency of Th1 cells and a reciprocal decrease in that of LAG3^+^ Tregs at day 7. Both Th1 cells and LAG3^+^ Tregs recovered their baseline percentages at day 14 (Fig. 5). In a former report, influenza vaccine administration did not influence the number of total CD4^+^CD25^+^ T cells, and it was supposed that the response to vaccine depends on the activation of different subsets of CD4^+^CD25^+^ T cells [28]. Active immunization may reduce the frequency of LAG3^+^ Tregs and increase that of Th1 cells to enhance immune responses.

## Discussion

We detected the human counterpart of murine LAG3^+^ Tregs [23] that produce high amounts of IL-10 and IFN-γ and strongly suppress B-cell antibody production. Although there are several reports stating that T cells suppress B-cell antibody production, most of them are related to CD25^+^ Tregs [29–31]. We found that human LAG3^+^ Tregs suppress B-cell antibody production more strongly than CD25^+^ Tregs. On one hand, an experiment using Transwell plates revealed that cell-to-cell contact played a great role in B-cell suppression by LAG3^+^ Tregs and that Fas–Fas ligand interaction might be involved in the mechanism. On the other hand, because LAG3^+^ Tregs suppressed B-cell antibody production to a certain extent even if they were separated from B and T_FH_ cells, it is difficult to deny the effect of some cytokines. One possible cytokine is transforming growth factor (TGF)-β3 because it is highly expressed in human LAG3^+^ Tregs, and murine LAG3^+^ Tregs directly suppress B-cell activation through TGF-β3 [23].

Regardless of CD49b expression, LAG3^+^ cells produced high amounts of IL-10 and suppressed B-cell antibody production, although Gagliani et al. concluded that coexpression of LAG3 and CD49b was necessary to identify human IL-10-producing T cells [22]. The difference may be due to the very low frequency of CD4^+^CD25^−^LAG3^+^CD49b^+^ T cells observed in our study. Although the role of CD49b should be investigated further, our results confirmed that LAG3 is a unique marker for IL-10-producing CD4^+^ T cells.

On one hand, in the patients with untreated RA, the frequency of CD25^+^ Tregs was not different from that observed in healthy donors, consistent with previous reports [32–34]. On the other hand, the frequency of LAG3^+^ Tregs was significantly lower in patients with RA than in healthy donors, and it was particularly low in patients with RA with higher disease activity. These results imply that LAG3^+^ Tregs are involved in regulating the pathophysiology of RA. Among several possible mechanisms, one is suppression of B-cell functions. Autoreactive B cells not only produce autoantibody that can potentiate immune responses but also can directly interact with T cells by antigen presentation, leading to cytokine production and germinal center formation [1, 35]. A pathogenic role for B cells other than antibody production was confirmed by the efficacy of rituximab with limited reduction of ACPA titers in RA [36]. The other possibility is that LAG3^+^ Tregs regulate RA via IL-10 production. IL-10-producing Tregs ameliorate collagen-induced arthritis in mice [37]. Moreover, Th17 cells play crucial roles in the development of RA [38], and IL-10-producing Tregs control inflammation by Th17 cells in an IL-10-dependent manner [39].

Administration of the costimulatory inhibitor abatacept significantly increased the frequency of LAG3^+^ Tregs in patients with RA. Moreover, naive CD4^+^ T cells stimulated in the presence of abatacept developed into CD4^+^ T cells with LAG3^+^ Treg-like manifestations. Although it is difficult to confirm the relationship between abatacept and LAG3^+^ Tregs, the expression of EGR2 strongly suggests the induction of LAG3^+^ Treg-like cells. The transcription factor EGR2 is identified as a T-cell anergy-associated gene [24] that is induced by TCR stimulation in the absence of costimulation. Moreover, EGR2 is highly expressed in both murine and human LAG3^+^ Tregs [21, 23]. Because abatacept binds to B7 molecules on APCs and inhibits T-cell costimulation [40], there is a possibility that abatacept enhances EGR2 expression by CD4^+^ T cells and increases the number of LAG3^+^ Tregs. Moreover, there was a significant increase in *FAS* expression and the tendency of *CD274* (*PDL1*) expression to increase in the abatacept-treated cells. Because Fas [23] and PD-L1 [27] were reported to play a role in the suppression ability of LAG3^+^ Tregs, there is a possibility that abatacept-treated cells exert suppressive ability using FAS or PD-L1 in the same way as naturally occurring LAG3^+^ Tregs.

Immunological stimulation such as autoimmunity and vaccination may reduce the number of LAG3^+^ Tregs via costimulation. LAG3^+^ Tregs could participate in the anti-inflammatory and immune-modulating effects achieved by a targeting therapy for costimulation. Actually, abatacept administration in patients with RA reduced the serum levels of immunoglobulins, ACPA, and RF as well as the percentage of postswitch memory B cells [41]. Increases in LAG3^+^ Tregs by abatacept treatment may be associated with control of humoral immunity. The fact that abatacept induces a general decrease of CD25^+^ Tregs is not consistent with the clinical efficacy of abatacept [42]. Increases of LAG3^+^ Tregs may compensate for the reduction of CD25^+^ Tregs in abatacept-treated patients.

With regard to the mechanisms of the suppressive ability of LAG3^+^ Tregs, multiple systems have been reported to be involved. In the present study, we revealed the importance of cell-cell contact and Fas. In vivo experiments using Fas-mutant B6/*lpr* mice confirmed that Fas expressed on LAG3^+^ Tregs plays a critical role in suppressing B-cell antibody production [23], and our results suggest that Fas could contribute to LAG3^+^ Treg suppressive ability in humans. However, our Transwell experiment also suggested that soluble factors have some effect because LAG3^+^ Treg suppression was still observed even if cell-to-cell contact was lost. One candidate is TGF-β3, which is highly expressed by both murine and human LAG3^+^ Tregs [43]. In vivo experiments confirmed that TGF-β3 strongly ameliorates B-cell-mediated pathology [23] and that TGF-β3 suppresses human B-cell differentiation into plasmablasts and antibody secretion [44]. We suppose that multiple mechanisms, including soluble factors (IL-10, TGF-β3) and cell-to-cell contact (PD-L1 and Fas), work together in bringing about LAG3^+^ Treg suppression.

In a previous report, Egr2-transduced LAG3^+^ Treg-like CD4^+^ T cells exhibited antigen-specific immunosuppressive capacity in vivo. On the basis of these findings, human LAG3^+^ Tregs are supposed to be specific to self-antigens associated with autoimmune disease; however, proving antigen specificity in human Tregs is difficult and remains to be elucidated.

Collectively, the results of our study suggest that human IL-10-producing-LAG3^+^ Tregs regulate immune responses related to the pathogenesis of RA. The induction of LAG3^+^ Tregs by abatacept implies a previously unrecognized role of IL-10-producing Tregs in the mode of action of abatacept.

## Conclusions

IL-10-producing LAG3^+^ Tregs in human PBMCs suppress the antibody production of B cells independent of IL-10. The frequency of LAG3^+^ Tregs is decreased in patients with RA and significantly increased with abatacept treatment. Abatacept-treated CD4^+^ T cells exhibit LAG3^+^ Treg-like function.

## Additional file

Additional file 1: Supplementary methods and figures. **Figure S1** Expression of FOXP3 in LAG3^+^ Tregs and CD25^+^ Tregs. Freshly isolated human CD4^+^ T cells were stained for FOXP3 and LAG3. Representative FACS data from three independent experiments are shown. **Figure S2** In vitro T-cell proliferation assay. Carboxyfluorescein succinimidyl ester-labeled naive T cells were cultured with irradiated APCs and CD25^+^ Tregs for 72 h. Representative FACS data from three independent experiments are shown. **Figure S3** Frequencies of IL-10^+^ cells in 7-AAD^−^ cells detected in intracellular staining of naive CD4^+^ T cells and LAG3^+^ Tregs (*n* = 4). * *P* < 0.05 by Mann-Whitney *U* test. **Figure S4** Scatterplot of frequencies of CD25^+^ Tregs or LAG3^+^ Tregs vs ACPA titer or RF (*n* = 83). *P* value is for Spearman’s rank correlation coefficient. *rho* Spearman’s rho. Linear regression line is drawn as a *blue line*, and 95% CI region is presented as a *gray area*. **a** Scatterplot of frequencies of CD25^+^ Tregs vs ACPA titer. **b** Scatterplot of frequencies of CD25^+^ Tregs vs RF titer. **c** Scatterplot of frequencies of LAG3^+^ Tregs vs ACPA titer. **d** Scatterplot of frequencies of LAG3^+^ Tregs vs RF titer. **Figure S5** Changes in percentages of LAG3^+^ Tregs in CD4^+^ T cells (ΔLAG3) were evaluated in accordance with treatment response to abatacept (*n* = 18). Patients were divided into three groups (no response, moderate response, and good response) following European League Against Rheumatism response criteria based on DAS28-ESR. * *P* < 0.05 by Kruskal-Wallis test and Dunn’s multiple-comparisons test. (ZIP 963 kb)

## Abbreviations

7-AAD: 7-Amino actinomycin D
Ab: Antibody
ACPA: Anticitrullinated protein antibody
APC: Antigen-presenting cell
CCP: Cyclic citrullinated peptide
CCR6: C-C chemokine receptor type 6
CCR7: C-C chemokine receptor type 7
CD: Cluster of differentiation
CDAI: Clinical Disease Activity Index
CRP: C-reactive protein
CXCR3: C-X-C chemokine receptor type 3
CXCR5: C-X-C chemokine receptor type 5
DAS28: Disease Activity Score in 28 joints
Egr2: Early growth response gene 2
ELISA: Enzyme-linked immunosorbent assay
ESR: Erythrocyte sedimentation rate
FACS: Fluorescence-activated cell sorting
FasL: Fas ligand
Foxp3: Forkhead box P3
*GAPDH*: Glyceraldehyde 3-phosphate dehydrogenase
HD: Healthy donor
IFN-γ: Interferon-γ
Ig: Immunoglobulin
IL: Interleukin
LAG3: Lymphocyte activation gene 3
mAb: Monoclonal antibody
mRNA: Messenger RNA
MTX: Methotrexate
PBMC: Peripheral blood mononuclear cell
PCR: Polymerase chain reaction
PD-L1: Programmed death-ligand 1
PE: Phycoerythrin
PerCP: Peridinin-chlorophyll protein
RA: Rheumatoid arthritis
RF: Rheumatoid factor
RPMI: Roswell Park Memorial Institute medium
SEB: Staphylococcal enterotoxin B
TCR: T-cell receptor
T_FH_: Follicular helper T cell
TGF-β: Transforming growth factor-β
Th1: Type 1 helper T cell
Th17: T helper 17 cell
TNF-α: Tumor necrosis factor-α
Treg: Regulatory T cell
