# Supplementary figures and images for: Interleukin-10-producing LAG3+ regulatory T cells are associated with disease activity and abatacept treatment in rheumatoid arthritis
Source: Arthritis Res Ther. 2017 May 16;19:97. doi: 10.1186/s13075-017-1309-x (PMC5434528; doi:10.1186/s13075-017-1309-x)

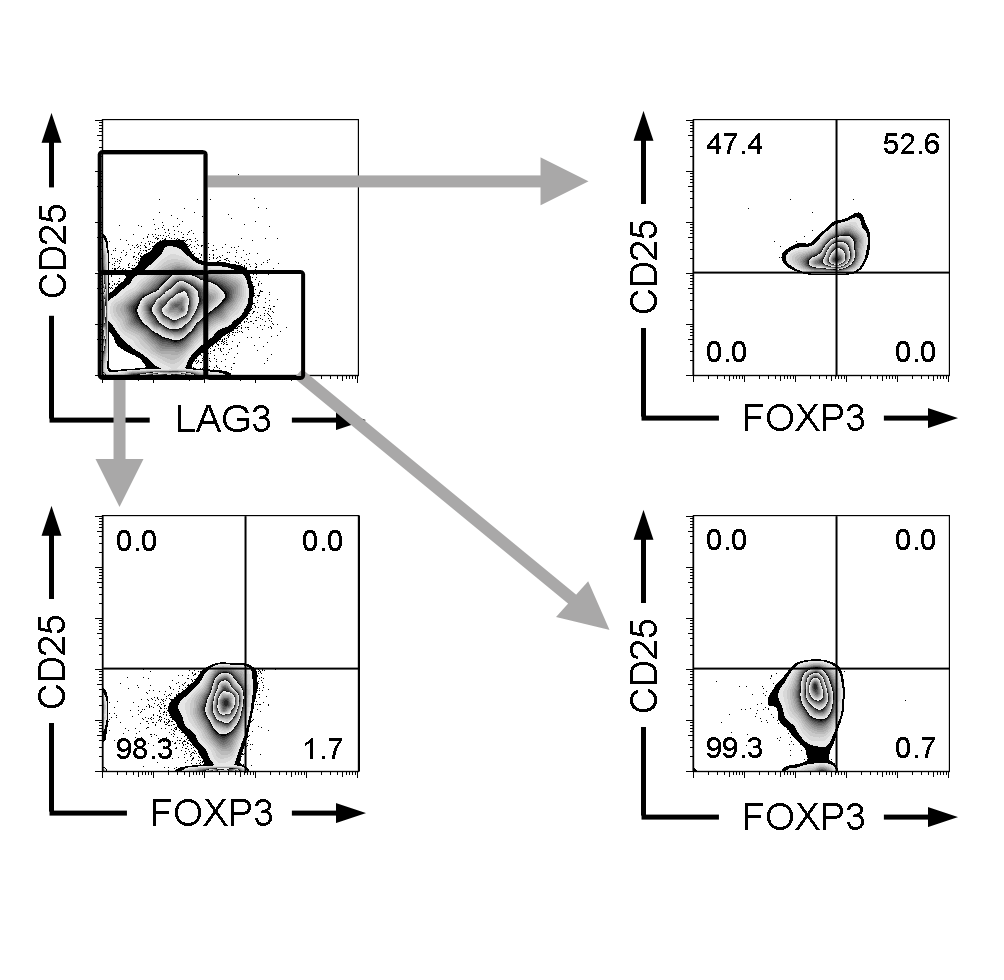

Supplement: Additional file 1: — Supplementary methods and figures. Figure S1 Expression of FOXP3 in LAG3+ Tregs and CD25+ Tregs. Freshly isolated human CD4+ T cells were stained for FOXP3 and LAG3. Representative FACS data from three independent experiments are shown. Figure S2 In vitro T-cell proliferation assay. Carboxyfluorescein succinimidyl ester-labeled naive T cells were cultured with irradiated APCs and CD25+ Tregs for 72 h. Representative FACS data from three independent experiments are shown. Figure S3 Frequencies of IL-10+ cells in 7-AAD− cells detected in intracellular staining of naive CD4+ T cells and LAG3+ Tregs (n = 4). * P < 0.05 by Mann-Whitney U test. Figure S4 Scatterplot of frequencies of CD25+ Tregs or LAG3+ Tregs vs ACPA titer or RF (n = 83). P value is for Spearman’s rank correlation coefficient. rho Spearman’s rho. Linear regression line is drawn as a blue line, and 95% CI region is presented as a gray area. a Scatterplot of frequencies of CD25+ Tregs vs ACPA titer. b Scatterplot of frequencies of CD25+ Tregs vs RF titer. c Scatterplot of frequencies of LAG3+ Tregs vs ACPA titer. d Scatterplot of frequencies of LAG3+ Tregs vs RF titer. Figure S5 Changes in percentages of LAG3+ Tregs in CD4+ T cells (ΔLAG3) were evaluated in accordance with treatment response to abatacept (n = 18). Patients were divided into three groups (no response, moderate response, and good response) following European League Against Rheumatism response criteria based on DAS28-ESR. * P < 0.05 by Kruskal-Wallis test and Dunn’s multiple-comparisons test. (ZIP 963 kb) [file 13075_2017_1309_MOESM1_ESM.zip › Figure S1.tif]

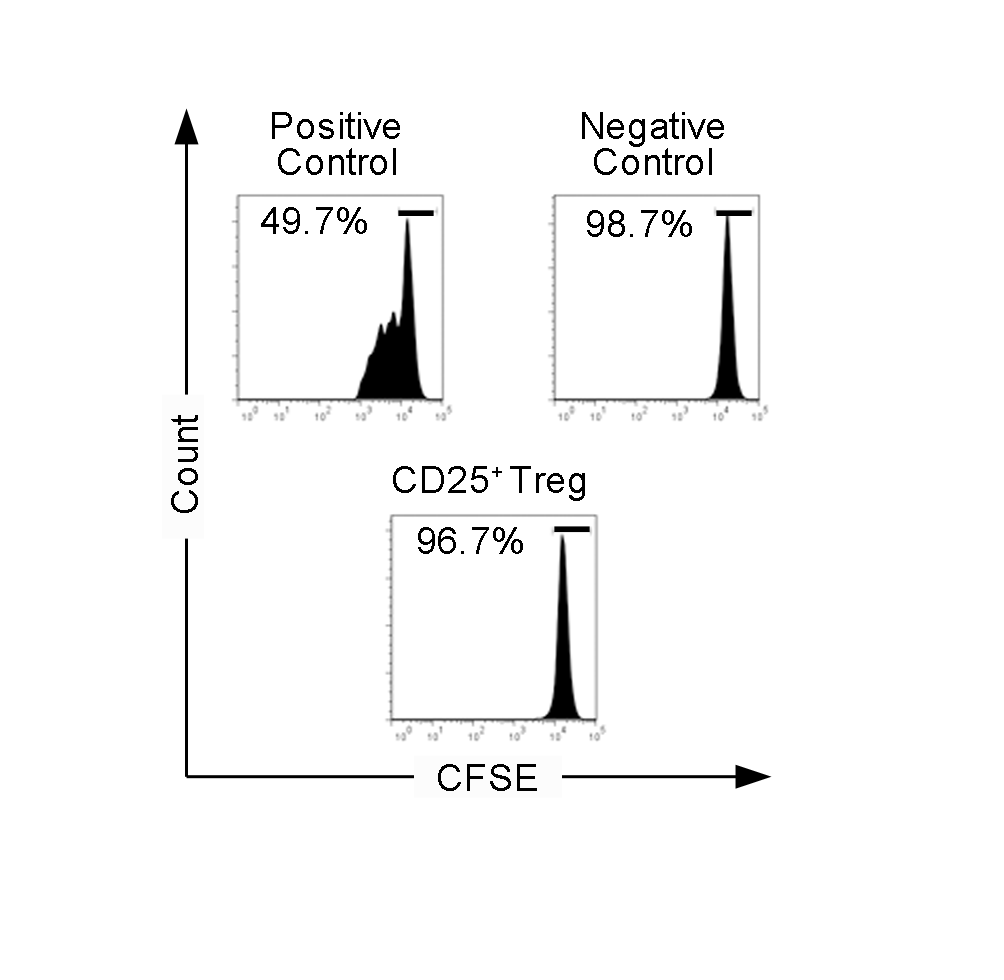

Supplement: Additional file 1: — Supplementary methods and figures. Figure S1 Expression of FOXP3 in LAG3+ Tregs and CD25+ Tregs. Freshly isolated human CD4+ T cells were stained for FOXP3 and LAG3. Representative FACS data from three independent experiments are shown. Figure S2 In vitro T-cell proliferation assay. Carboxyfluorescein succinimidyl ester-labeled naive T cells were cultured with irradiated APCs and CD25+ Tregs for 72 h. Representative FACS data from three independent experiments are shown. Figure S3 Frequencies of IL-10+ cells in 7-AAD− cells detected in intracellular staining of naive CD4+ T cells and LAG3+ Tregs (n = 4). * P < 0.05 by Mann-Whitney U test. Figure S4 Scatterplot of frequencies of CD25+ Tregs or LAG3+ Tregs vs ACPA titer or RF (n = 83). P value is for Spearman’s rank correlation coefficient. rho Spearman’s rho. Linear regression line is drawn as a blue line, and 95% CI region is presented as a gray area. a Scatterplot of frequencies of CD25+ Tregs vs ACPA titer. b Scatterplot of frequencies of CD25+ Tregs vs RF titer. c Scatterplot of frequencies of LAG3+ Tregs vs ACPA titer. d Scatterplot of frequencies of LAG3+ Tregs vs RF titer. Figure S5 Changes in percentages of LAG3+ Tregs in CD4+ T cells (ΔLAG3) were evaluated in accordance with treatment response to abatacept (n = 18). Patients were divided into three groups (no response, moderate response, and good response) following European League Against Rheumatism response criteria based on DAS28-ESR. * P < 0.05 by Kruskal-Wallis test and Dunn’s multiple-comparisons test. (ZIP 963 kb) [file 13075_2017_1309_MOESM1_ESM.zip › Figure S2.tif]

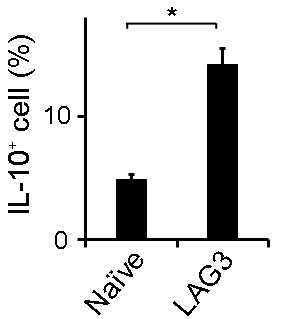

Supplement: Additional file 1: — Supplementary methods and figures. Figure S1 Expression of FOXP3 in LAG3+ Tregs and CD25+ Tregs. Freshly isolated human CD4+ T cells were stained for FOXP3 and LAG3. Representative FACS data from three independent experiments are shown. Figure S2 In vitro T-cell proliferation assay. Carboxyfluorescein succinimidyl ester-labeled naive T cells were cultured with irradiated APCs and CD25+ Tregs for 72 h. Representative FACS data from three independent experiments are shown. Figure S3 Frequencies of IL-10+ cells in 7-AAD− cells detected in intracellular staining of naive CD4+ T cells and LAG3+ Tregs (n = 4). * P < 0.05 by Mann-Whitney U test. Figure S4 Scatterplot of frequencies of CD25+ Tregs or LAG3+ Tregs vs ACPA titer or RF (n = 83). P value is for Spearman’s rank correlation coefficient. rho Spearman’s rho. Linear regression line is drawn as a blue line, and 95% CI region is presented as a gray area. a Scatterplot of frequencies of CD25+ Tregs vs ACPA titer. b Scatterplot of frequencies of CD25+ Tregs vs RF titer. c Scatterplot of frequencies of LAG3+ Tregs vs ACPA titer. d Scatterplot of frequencies of LAG3+ Tregs vs RF titer. Figure S5 Changes in percentages of LAG3+ Tregs in CD4+ T cells (ΔLAG3) were evaluated in accordance with treatment response to abatacept (n = 18). Patients were divided into three groups (no response, moderate response, and good response) following European League Against Rheumatism response criteria based on DAS28-ESR. * P < 0.05 by Kruskal-Wallis test and Dunn’s multiple-comparisons test. (ZIP 963 kb) [file 13075_2017_1309_MOESM1_ESM.zip › Figure S3.tif]

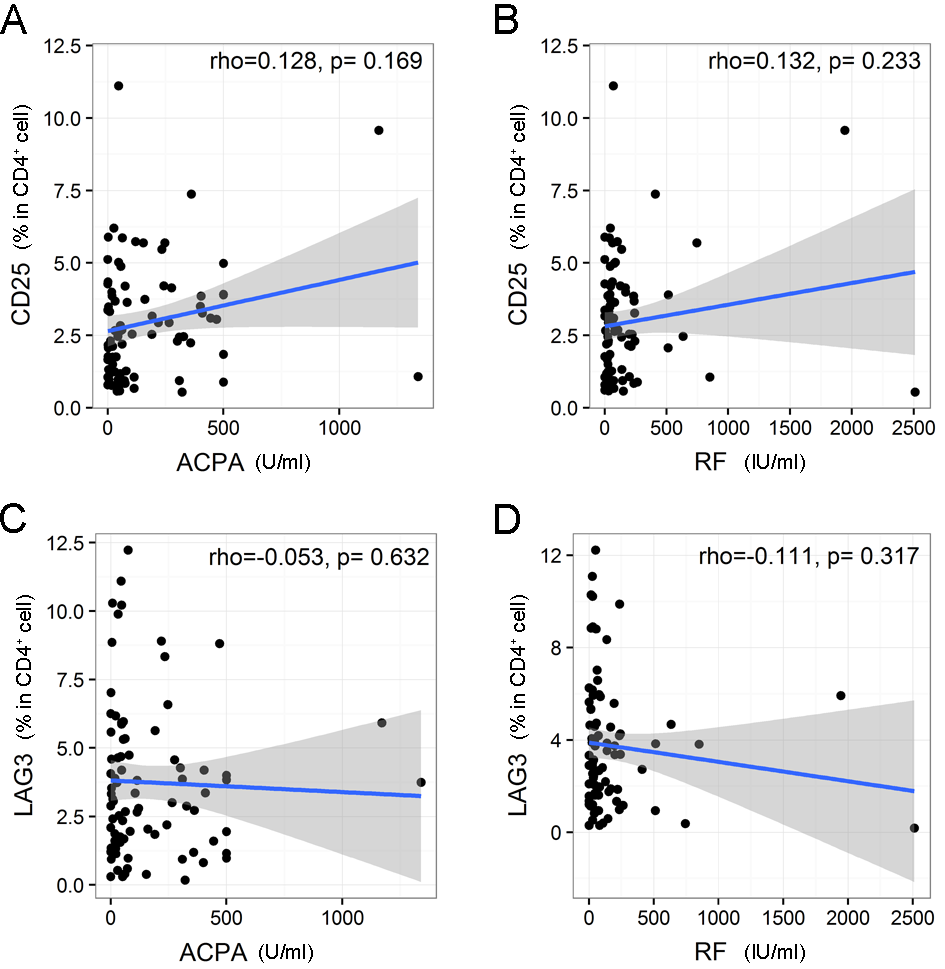

Supplement: Additional file 1: — Supplementary methods and figures. Figure S1 Expression of FOXP3 in LAG3+ Tregs and CD25+ Tregs. Freshly isolated human CD4+ T cells were stained for FOXP3 and LAG3. Representative FACS data from three independent experiments are shown. Figure S2 In vitro T-cell proliferation assay. Carboxyfluorescein succinimidyl ester-labeled naive T cells were cultured with irradiated APCs and CD25+ Tregs for 72 h. Representative FACS data from three independent experiments are shown. Figure S3 Frequencies of IL-10+ cells in 7-AAD− cells detected in intracellular staining of naive CD4+ T cells and LAG3+ Tregs (n = 4). * P < 0.05 by Mann-Whitney U test. Figure S4 Scatterplot of frequencies of CD25+ Tregs or LAG3+ Tregs vs ACPA titer or RF (n = 83). P value is for Spearman’s rank correlation coefficient. rho Spearman’s rho. Linear regression line is drawn as a blue line, and 95% CI region is presented as a gray area. a Scatterplot of frequencies of CD25+ Tregs vs ACPA titer. b Scatterplot of frequencies of CD25+ Tregs vs RF titer. c Scatterplot of frequencies of LAG3+ Tregs vs ACPA titer. d Scatterplot of frequencies of LAG3+ Tregs vs RF titer. Figure S5 Changes in percentages of LAG3+ Tregs in CD4+ T cells (ΔLAG3) were evaluated in accordance with treatment response to abatacept (n = 18). Patients were divided into three groups (no response, moderate response, and good response) following European League Against Rheumatism response criteria based on DAS28-ESR. * P < 0.05 by Kruskal-Wallis test and Dunn’s multiple-comparisons test. (ZIP 963 kb) [file 13075_2017_1309_MOESM1_ESM.zip › Figure S4.tif]

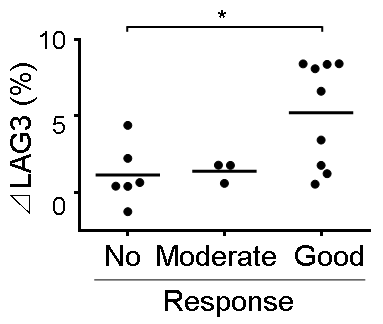

Supplement: Additional file 1: — Supplementary methods and figures. Figure S1 Expression of FOXP3 in LAG3+ Tregs and CD25+ Tregs. Freshly isolated human CD4+ T cells were stained for FOXP3 and LAG3. Representative FACS data from three independent experiments are shown. Figure S2 In vitro T-cell proliferation assay. Carboxyfluorescein succinimidyl ester-labeled naive T cells were cultured with irradiated APCs and CD25+ Tregs for 72 h. Representative FACS data from three independent experiments are shown. Figure S3 Frequencies of IL-10+ cells in 7-AAD− cells detected in intracellular staining of naive CD4+ T cells and LAG3+ Tregs (n = 4). * P < 0.05 by Mann-Whitney U test. Figure S4 Scatterplot of frequencies of CD25+ Tregs or LAG3+ Tregs vs ACPA titer or RF (n = 83). P value is for Spearman’s rank correlation coefficient. rho Spearman’s rho. Linear regression line is drawn as a blue line, and 95% CI region is presented as a gray area. a Scatterplot of frequencies of CD25+ Tregs vs ACPA titer. b Scatterplot of frequencies of CD25+ Tregs vs RF titer. c Scatterplot of frequencies of LAG3+ Tregs vs ACPA titer. d Scatterplot of frequencies of LAG3+ Tregs vs RF titer. Figure S5 Changes in percentages of LAG3+ Tregs in CD4+ T cells (ΔLAG3) were evaluated in accordance with treatment response to abatacept (n = 18). Patients were divided into three groups (no response, moderate response, and good response) following European League Against Rheumatism response criteria based on DAS28-ESR. * P < 0.05 by Kruskal-Wallis test and Dunn’s multiple-comparisons test. (ZIP 963 kb) [file 13075_2017_1309_MOESM1_ESM.zip › Figure S5.tif]
