# Supplementary material for: Interleukin-10-producing LAG3+ regulatory T cells are associated with disease activity and abatacept treatment in rheumatoid arthritis
Source: Arthritis Res Ther. 2017 May 16;19:97. doi: 10.1186/s13075-017-1309-x (PMC5434528; doi:10.1186/s13075-017-1309-x)
Supplement: Additional file 1: — Supplementary methods and figures. Figure S1 Expression of FOXP3 in LAG3+ Tregs and CD25+ Tregs. Freshly isolated human CD4+ T cells were stained for FOXP3 and LAG3. Representative FACS data from three independent experiments are shown. Figure S2 In vitro T-cell proliferation assay. Carboxyfluorescein succinimidyl ester-labeled naive T cells were cultured with irradiated APCs and CD25+ Tregs for 72 h. Representative FACS data from three independent experiments are shown. Figure S3 Frequencies of IL-10+ cells in 7-AAD− cells detected in intracellular staining of naive CD4+ T cells and LAG3+ Tregs (n = 4). * P < 0.05 by Mann-Whitney U test. Figure S4 Scatterplot of frequencies of CD25+ Tregs or LAG3+ Tregs vs ACPA titer or RF (n = 83). P value is for Spearman’s rank correlation coefficient. rho Spearman’s rho. Linear regression line is drawn as a blue line, and 95% CI region is presented as a gray area. a Scatterplot of frequencies of CD25+ Tregs vs ACPA titer. b Scatterplot of frequencies of CD25+ Tregs vs RF titer. c Scatterplot of frequencies of LAG3+ Tregs vs ACPA titer. d Scatterplot of frequencies of LAG3+ Tregs vs RF titer. Figure S5 Changes in percentages of LAG3+ Tregs in CD4+ T cells (ΔLAG3) were evaluated in accordance with treatment response to abatacept (n = 18). Patients were divided into three groups (no response, moderate response, and good response) following European League Against Rheumatism response criteria based on DAS28-ESR. * P < 0.05 by Kruskal-Wallis test and Dunn’s multiple-comparisons test. (ZIP 963 kb) [file 13075_2017_1309_MOESM1_ESM.zip › Additional file 1.doc]

**Supplementary methods:**

**T cell suppression assay**

Sorted naïve CD4^+^ T cells (3 × 10^4^ cells/well) were labeled with carboxyfluorescein diacetate succinimidyl ester (CFSE; Dojindo) and cultured with APCs (1 × 10^5^ cells/well) and CD25^+^ Treg (3 × 10^4^ cells/well) with anti-CD3 Ab (5.0 μg/mL). APCs were sorted as CD3-negative cells and irradiated with 30 Gy. Cells cultured in the prepared medium as shown in the main article were sorted as 7-amino-actinomycin-D-negative cells (7-AAD; BioLegend) and analyzed at day 3.

**Intracellular staining for FOXP3 expression.**

PBMCs were isolated from human healthy controland superficially stained in the same manner as other experiments. A labeling procedure for FOXP3 was conducted, followed by fixation and permeabilization with FITC anti-FOXP3 Ab (PCH101, from eBioscience).

**Intracellular staining for IL-10 expression.**

Naïve CD4^+^ T cells and LAG3^+^ Tregs were sorted and stimulated for 72 hours with anti-CD3ε mAb (10 μg/ml) and anti-human CD28 mAb (5 μg/ml) in the presence of recombinant human IL-2 (100 IU/ml). 24 hours prior to cytokine production analysis, phorbol 12-myristate 13-acetate (PMA) (25 ng/ml), ionomycin (1 μg/ml) and protein transport inhibitor GolgiStop (BD) were added. After staining with 7-AAD, intracellular staining was performed using Cytofix/Cytoperm Fixation/Permeabilization Kit (BD) following manufacturer’s instruction. For cytokine production analysis, IL-10-Alexa Fluor 660 (JES3-9D7, eBioscience) antibodies were used.

**Supplementary figures:**

**Supplementary figure S1** Expression of FOXP3 in LAG3^+^ Treg and CD25^+^ Treg. Freshly isolated human CD4^+^ T cells were stained for FOXP3 and LAG3. Representative FACS data from three independent experiments are shown.

**Supplementary figure S2** *In vitro* T cell proliferation assay. CFSE-labeled naïve T cells were cultured with irradiated antigen presenting cells (APCs) and CD25^+^ Tregs for 72 hours. Representative FACS data from three independent experiments are shown.

**Supplementary figure S3** Frequencies of IL-10^+^ cells in 7AAD^-^ cells detected in intracellular staining of naïve CD4^+^ T cells and LAG3^+^ Treg (n = 4). * *P* < 0.05; Mann-Whitney U test.

**Supplementary figure S4** Scatter plot of frequencies of CD25^+^ Treg or LAG3^+^ Treg versus anti-CCP antibody (ACPA) titer or rheumatoid factor (RF) (n=83). rho; Spearman’s rho. p; p-value of Spearman's rank correlation coefficient. Linear regression line is drawn as a blue line, and 95% confidence region is presented as a grey area. (A) Scatter plot of frequencies of CD25^+^ Treg versus ACPA titer. (B) Scatter plot of frequencies of CD25^+^ Treg versus RF titer. (C) Scatter plot of frequencies of LAG3^+^ Treg versus ACPA titer. (D) Scatter plot of frequencies of LAG3^+^ Treg versus RF titer.

**Supplementary figure S5** Changes of percentages of LAG3^+^ Tregs in CD4^+^ T cells (ΔLAG3) were evaluated in accordance with treatment response to abatacept (n = 18). Patients were divided into three groups (no response, moderate response, good response) following EULAR response criteria based on DAS28-ESR. *, P < 0.05; Kruskal-Wallis test and Dunn’s multiple comparison test.
